# Ultrasound‐Guided Airway Nerve Blocks vs. Lidocaine Topical Anesthesia Using Video‐Assisted Laryngoscopy in Suspected Difficult Intubation for Patients Undergoing Bariatric Surgery: A Randomized Clinical Trial

**DOI:** 10.1155/anrp/2054379

**Published:** 2026-05-22

**Authors:** Reham M. Hashim, Mohamed S. M. Zaki, David B. N. Elagiby, Islam A. A. Taher

**Affiliations:** ^1^ Anesthesia, ICU, and Pain Management, Faculty of Medicine, Ain Shams University, Cairo, 11591, Egypt, asu.edu.eg

**Keywords:** airway nerve block, difficult intubation, topical anesthesia

## Abstract

**Introduction:**

Anesthetic complications related to difficult airways in obese patients are very common, necessitating the need for an ideal method of awake intubation, either airway block anesthesia or topical anesthesia.

**The Aim of This Work:**

To compare topical airway anesthesia with airway nerve block for ease of awake intubation, with the C‐MAC video laryngoscope device employed in difficult intubation anticipated in patients undergoing bariatric surgery. Ease of intubation has been assessed by comparing gag reflex, cough reflex, patient comfort, and other factors.

**Patients and Methods:**

This study has been conducted on 80 obese patients undergoing bariatric surgery, where 40 patients received airway nerve block Group LA and 40 patients received topical anesthesia by the method of spray as you go for anesthetizing the airway using 10% lidocaine spray Group *T*, in addition to conscious sedation before video‐assisted laryngoscopy.

**Result:**

Group LA showed better airway conditions with decreased cough and gag reflexes, and more patients were intubated at the first attempt than in Group *T*. Intubation time and need for a rescue airway were less in Group LA. Patient comfort and experience, by recall absence and lower Visual Analog Scale (VAS) scores, were better for Group LA in comparison with Group *T*. Adverse events in terms of heart rate (HR), elevated mean blood pressure (MAP), and desaturation episodes were more common in Group *T*.

**Conclusion:**

This study concluded that ultrasound‐guided airway nerve blocks provided shorter intubation time, significantly superior airway conditions, and more stable hemodynamic and oxygenation profiles compared to topical lidocaine spray, with fewer airway‐related complications, reduced need for rescue interventions, and higher overall comfort with no recall of the procedure.

**Trial Registration:** ClinicalTrials.gov identifier: NCT07069686

## 1. Introduction

One of the most significant public health problems across the world is obesity. Quality of life can be improved, sustained weight loss can be provided, and several obesity‐related comorbidities can be remitted through bariatric surgery, which is an efficient treatment for severe obesity [1]. 40% of adverse events related to airway complications are mainly caused by obesity [2]. Difficult mask ventilation and difficult laryngoscopy are anesthetic complications that can lead to severe mortality and morbidity, resulting in elevated risks of an unsuccessful result and a surgical failure [3].

Anesthetizing the airway is performed via two main methods: the first involves injecting local anesthesia (LA) for blocking airway nerves, and the second is topical application comprising nebulization, atomization, or the “spray as you go” technique (this is the method that this study uses) [4].

Superior laryngeal nerve (SLN) blocks with translaryngeal injection and bilateral glossopharyngeal nerve blocks are required for full upper airway anesthesia [5]. Motor, parasympathetic, and sensory fibers are included in the glossopharyngeal nerve [6]. The vagus nerve is the source of the SLN. Sensory function is supplied via the internal branch to the pharynx’s lower part and the larynx’s upper portion above the vocal cords (tongue base, arytenoids, aryepiglottic folds, and epiglottis) [7]. As a branch of the vagus nerve, the recurrent laryngeal nerve is primarily related to the motor innervation of the laryngeal intrinsic muscles [8]. To ensure successful instrumentation and the awake patient’s comfort, airway topical anesthesia has to be effectively performed [9].

### 1.1. Aim of the Work

Using the C‐MAC video laryngoscope device, this study compares the airway nerve blocks to the topical lidocaine spray as‐you‐go technique for ease of awake intubation regarding anticipated difficult intubation in patients going through bariatric surgery. Ease of intubation is assessed by comparing the gag reflex, cough reflex, patient comfort, and other factors.

### 1.2. Patients and Methods

This study received approval from the ethical committee at the Faculty of Medicine, Ain Shams University (FMASU MS 120/2025), after obtaining informed written consents from 80 patients that the study included in a randomized single‐blinded clinical trial following the CONSORT 2010 guidelines. The study was conducted at the operating theaters of Ain Shams University over 6 months. Obese patients between 21 and 65 years old, with a BMI > 35 kg/m^2,^ and going through bariatric surgery were chosen for the study. Those patients were ASA I–II with criteria of suspected difficult intubation, Mallampati score ≥ 3, thyromental distance (TMD) < 6 cm, limited neck mobility, and patients with a history of difficult intubation.

Patients facing heightened risk for aspiration (e.g., gastroesophageal reflux disease, full stomach, or pregnancy), expected difficult ventilation, who have upper airway pathology or surgery, with a mouth opening < 2 cm, ASA III–IV, and undergoing emergency surgery were excluded from the study. Also, patients allergic to local anesthetics, infection at the injection site, and coagulopathy, with neurological or cognitive impairment making the patient uncooperative, history of difficult airway complications, for example, previous intubation failure or need for surgical airway, and with cervical spine instability or fractures were also excluded.

### 1.3. Randomization Sampling Method

Employing computer‐generated randomization, randomly allocating patients into two groups: local anesthesia (LA) and topical (T) groups. Both the patients and the treating physician were aware of the treatment allocation, while the data collector and analyst remained blinded to the study procedure (single‐blinded study). To minimize assessment bias, cough and gag reflexes were evaluated by a designated independent observer (an anesthesia resident not involved in performing either airway technique), using prespecified binary criteria applied contemporaneously during the procedure. Patient comfort (Visual Analog Scale [VAS] score) and procedural recall were assessed postoperatively by the same independent observer through a structured patient interview in the recovery area.

Group LA (40): Patients were intubated using the video‐assisted laryngoscope after taking an airway nerve block guided by an ultrasound device.

Group *T* (40): Patients were intubated using the video‐assisted laryngoscope after topical lidocaine 10% spraying as‐you‐go to the oropharyngeal cavity without exceeding the toxic dose.

#### 1.3.1. Sample Size

Sample size was calculated based on the anticipated difference in proportions of patients experiencing a composite cough/gag reflex, using a two‐proportions *z*‐test (two‐sided) as implemented in PASS 15 software (NCSS, Kaysville, UT, USA). Based on Gupta et al. [10], the expected cough/gag reflex incidence was approximately 62% in Group *T* and 20% in Group LA. With alpha = 0.05 (two‐sided) and 80% power, a minimum of 36 patients per group was required. After a 10% dropout adjustment, 40 patients per group (80 total) were enrolled.

The single primary endpoint used for sample size calculation was the composite incidence of cough/gag reflex, defined as the presence of either or both reflexes during laryngoscopy and endotracheal tube placement.

A ≥ 42 percentage‐point absolute reduction in composite cough/gag reflex incidence was defined as the minimal clinically important difference MCID, as it represents a clinically meaningful transition from majority‐to‐minority airway reactivity with direct implications for intubation success, hemodynamic stability, and patient safety during awake difficult airway management.

### 1.4. Study Procedures

The study conducted a comprehensive preoperative evaluation, which comprised a full airway evaluation (body weight, mouth opening, Mallampati grading, dentition evaluation, and TMD). A prescription of anti‐aspiration prophylaxis, with 40 mg of Controloc, was administered with standard fasting guidelines. The awake video‐laryngoscopy intubation procedure was informed to patients during the preoperative assessment while administering 0.4 mg of atropine injection 30 min before transferring the patient to the operating room (OR) to decrease secretions and thereby ease intubation. Inside the OR, standard monitoring, including electrocardiography (ECG), noninvasive blood pressure (BP), and pulse oximetry (SpO2), was applied in all patients. An intravenous (IV) line was secured, and Ringer’s lactate solution was started.

After the baseline heart rate (HR), SpO_2_, and BP were recorded, dexmedetomidine 0.7 mcg/kg/hr infusion was given, and a single ketamine 0.5 mg/kg IV dose was injected for providing conscious sedation while performing the techniques and until the endotracheal tube was secured using a C‐MAC D‐Blade video laryngoscope (blade 3 or 4).

### 1.5. LA Group

**FIGURE 1:**
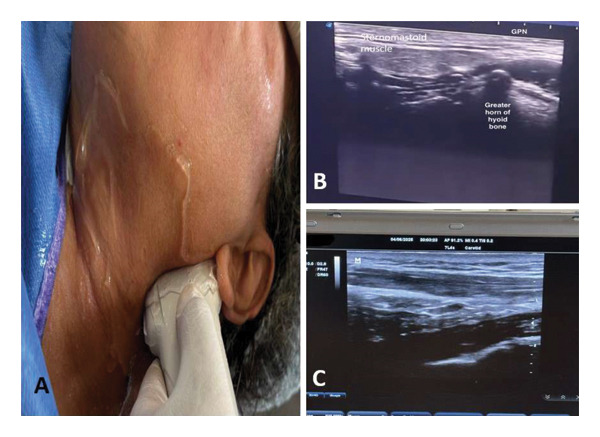
Glossopharyngeal nerve block using a parapharyngeal approach. (A) The anatomical placement of the probe. (B) The anatomical landmarks for the site of injection, including the greater horn of the hyoid bone. (C) The dispersion of the local anesthetic agent along the parapharyngeal space.

For performing the glossopharyngeal nerve block, the parapharyngeal space approach was utilized, employing 1.5 mL of 2% lidocaine. Utilizing the Sonosite turbo M (Bothell, Washington, USA) ultrasound machine, the patient was placed supine with the head tilted to the opposite side of the block. A linear ultrasound probe (6–15 MHz) was used and placed inferior to the mandibular angle. A cephalad tilting of the probe was performed for the pharyngeal wall to be located between the mandible and the greater horn of the hyoid bone, and then the pharyngeal wall close to the tonsil was targeted. Initially, the plan was needle insertion using the ultrasound probe in the right lateral edge with the in‐plane technique. Nevertheless, the proximal facial artery was near the intended puncture site. Hence, an out‐of‐plane technique was employed to change the puncture side to the superior side to avoid arterial injury. A 22–25 gauge needle was inserted, followed by aspiration to ensure the absence of any intravascular placement (Figure 1).

**FIGURE 2:**
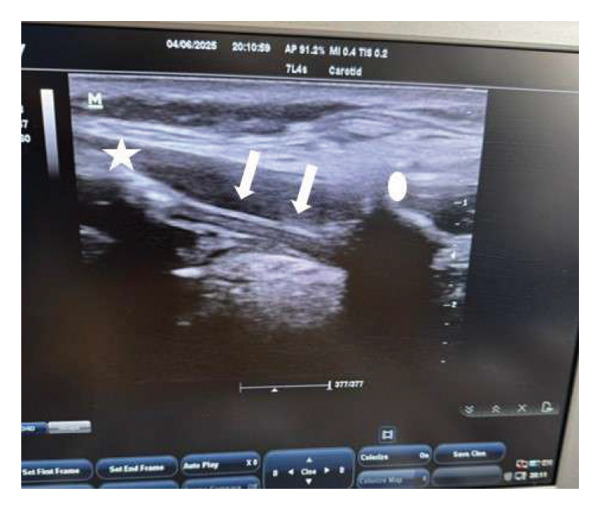
Superior laryngeal nerve block. An ultrasound image shows the anatomical landmarks for the injection site. The star sign indicates the hyoid bone, while the round sign indicates the thyroid cartilage. The arrows show the thyrohyoid membrane, which is the injection site.

The out‐of‐plane approach was employed to perform the bilateral SLN blocks. The patient was placed supine with the neck extended. A longitudinal/sagittal application of the linear probe in the submandibular area near the thyroid cartilage lateral edge was applied with medial orientation till identification of the hyoid bone cranially and the thyroid cartilage caudally, with the thyrohyoid membrane seen in between, offering an image that guides needle insertion. A 25‐G needle (25 mm) was used to inject 1 mL of 2% lidocaine into the thyrohyoid membrane (Figure 2). A downward displacement of the thyrohyoid membrane was considered a sign of successful injection.

**FIGURE 3:**
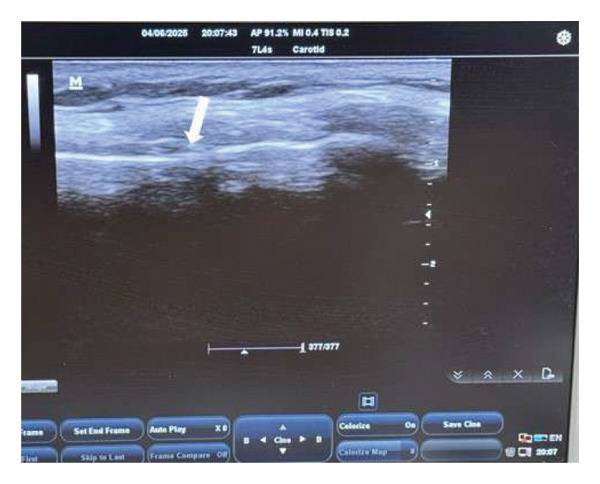
Recurrent laryngeal nerve block. An ultrasound image shows the anatomical landmarks for the injection site. The arrow shows the cricothyroid membrane, which is the injection site.

A translaryngeal out‐of‐plane approach was employed to perform bilateral recurrent laryngeal nerve blocks. First, the neck of the patient was placed in an extension position, and then the probe was placed in a transverse position to identify the thyroid cartilage, which appeared as a triangular roof. The probe was moved cranially till identification of the cricoid cartilage appeared as an arch‐shaped structure. In between, a hyperechoic bright horizontal line appeared in the midline, representing the cricothyroid membrane. Approximately 1.5 cm toward the cricothyroid membrane, a 25‐G 25‐mm needle was inserted with 2 mL 2% lidocaine at the cricothyroid membrane level after air backflow confirmation (Figure 3). This technique triggered the cough reflex, causing dispersion of the anesthetic agent to the vocal cords and the subglottic mucosa.

### 1.6. Topical Anesthesia Group

Patients received topical lidocaine 10% spray directly on the tongue and oropharynx without exceeding toxic doses (4.5 mg/kg max 300 mg; 10% lidocaine spray 10 g/100 mL = 100 mg/mL, so 1 spray is approximately 10 mg).

In Group LA patients, LA had been confirmed adequate through voice hoarseness, while Group *T* patients displayed tongue numbness. C‐MAC intubation was performed, inserting appropriate endotracheal tubes, while supplemental oxygen was given through nasal prongs. At 1 min and 3 min postintubation and during intubation, vital parameters (BP, SpO_2_, and HR) were also recorded. Seizures, bronchoconstriction, ECG changes, and any other lignocaine toxicity signs were also noted.

After securing the airway, general anesthesia was performed using sevoflurane inhalational anesthesia, rocuronium 0.6 mg/kg, and propofol 2 mg/kg. Then, patient comfort was assessed postoperatively for dysphagia, sore throat, voice change, or other gum, lip, dental, tongue injuries, partial recall, complete amnesia, and unpleasant memories during C‐MAC awake intubation.

### 1.7. Measurements

The single primary endpoint used for sample size calculation was the composite incidence of cough/gag reflex, defined as the presence of either or both reflexes during laryngoscopy and endotracheal tube placement.

Cough and gag reflexes were assessed using a prespecified binary scale (0 = *absent*; 1 = *present*). Cough reflex was defined as any involuntary expiratory muscular contraction triggered during laryngoscope insertion, endotracheal tube passage through the vocal cords, or cuff inflation. Gag reflex was defined as any visible pharyngeal or palatal retching movement elicited by contact of the laryngoscope blade with the posterior oropharyngeal wall or tongue base. Reflexes were assessed at three standardized time points: (i) laryngoscope insertion; (ii) endotracheal tube passage through the vocal cords; and (iii) cuff inflation. A composite present outcome was assigned if either reflex was observed at any time point. Assessments were performed by a designated independent observer (an anesthesia resident not involved in performing either airway technique) who received a standardized prestudy briefing on operational definitions and assessment timing. While a universally validated reflex scoring instrument does not exist for this clinical context, the binary methodology is consistent with comparable published trials [7, 10, 11]. Formal inter‐observer reliability testing was not performed.

Secondary outcomes involved time required for intubation, HR, oxygen saturation (SpO_2_), mean blood pressure (MAP), and intubation success (needing additional airway intervention, for example, supraglottic device, fiber optic, or surgical airway). Also, patient tolerance, comfort, and satisfaction via VAS (score 0 = *no discomfort* to 10 = *severe discomfort*), number of intubation attempts, and the need for rescue anesthesia (record if additional local anesthetics, sedation, or other airway management interventions were needed).

Complications, including laryngospasm, bronchospasm, aspiration, bleeding, postoperative sore throat, and local anesthetic toxicity, were recorded.

### 1.8. Statistical Analysis

Using Version 25.0 (IBM Corp., Armonk, NY, USA) of the Statistical Package for Social Sciences (SPSS) software, reviewing, coding, and analyzing the collected data were performed. Data presentation and analysis were performed according to the distribution and type of each variable.

Normality testing: Normality of numerical data distributions was assessed using the Shapiro–Wilk test. Descriptive statistics: The expression of numerical variables was given as mean ± standard deviation (SD), while summarizing categorical variables as percentages and frequencies. Analytical statistics: The means between two groups were compared using Student’s *t*‐test to reach normally distributed data. The two‐sided *z*‐test for proportions assumed in the sample size calculation is statistically equivalent to the chi‐square test used for binary outcome analysis (chi‐squared = z‐squared), ensuring methodological alignment between the power analysis assumptions and the inferential tests employed, consistent with CONSORT 2010 guidelines.

The chi‐square (*χ* ^2^) test was employed for examining associations between categorical variables. Regression Analysis: To identify binary outcomes’ predictors, logistic regression was employed. Results were reported as odds ratios (ORs) with 95% confidence intervals (CIs).

OR = 1 indicates no effect.

OR > 1 indicates elevated outcome odds (risk factor).

OR < 1 indicates reduced outcome odds (protective factor).

A narrower CI indicates greater precision in the estimate.

#### 1.8.1. Statistical Significance

The *p* value < 0.05 was regarded as significant, according to a 95% confidence level.

## 2. Results

**FIGURE 4:**
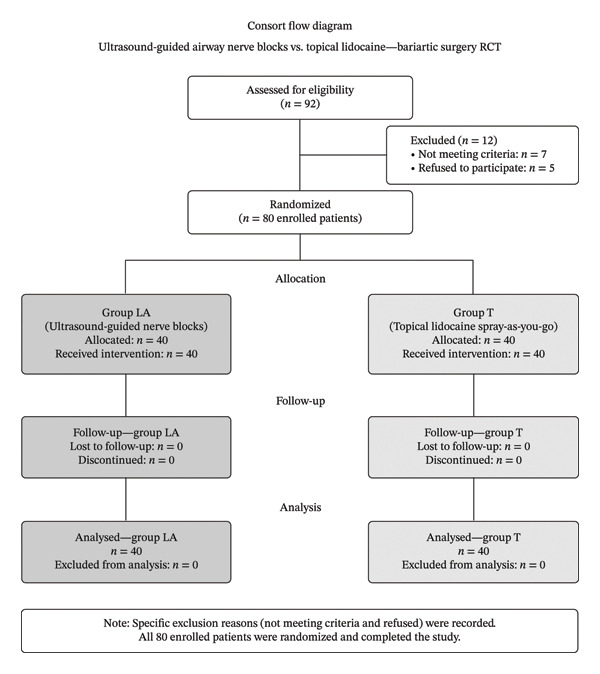
CONSORT flow diagram showing participant allocation and analysis.

This study was carried out on 80 obese patients with anticipated difficult intubation, undergoing bariatric surgery at Ain Shams University Hospitals, after the exclusion of 12 patients (Figure 4). Random allocation of participants into two equal groups was carried out: Group *T* (*n* = 40) and Group LA (*n* = 40).

According to the demographics data, no statistically significant differences were noted between Group *T* and Group LA regarding sex distribution, age, or BMI.

Regarding airway assessment, no observed significant differences were recorded between Group *T* and Group LA in Mallampati score or in TMD (Table 1).

**TABLE 1:** Airway assessment in the studied groups.

**Parameter**		**Group LA (*n* = 40)**	**Group *T* (*n* = 40)**	**p** **value**	**Significance**

Mallampati score	III	22 (55.0%)	23 (57.5%)	*χ* ^2^:0.000, *p* = 1.000	NS
	IV	18 (45.0%)	17 (42.5%)		

TMD (cm)	Mean ± SD	5.39 ± 0.34	5.38 ± 0.31	*t*:0.103, *p* = 0.918	NS

*Note: t*: *t*‐test; *χ* ^2^: chi‐square.

Abbreviations: NS, nonsignificance; SD: standard deviation.

Group LA had a significantly lower incidence of cough/gag reflex compared to Group *T*. Additionally, a higher percentage of patients in Group LA were intubated on the first attempt successfully (Table 2). Group LA had a significantly lower incidence of cough/gag reflex compared to Group *T* (20.0% vs. 62.5%; *p* < 0.001). The magnitude of this difference was clinically substantial: OR = 0.15 (95% CI: 0.055–0.410), indicating 85% lower odds of cough/gag reflex in Group LA; relative risk (RR) = 0.32 (95% CI: 0.165–0.622), reflecting a 68% RR reduction; and absolute risk reduction (ARR) = 42.5% (95% CI: 23.0%–62.0%), with a number needed to treat (NNT) of approximately 2.4. All CIs exclude the null value, confirming both statistical and clinical significance. Additionally, a higher percentage of patients in Group LA were successfully intubated on the first attempt. Concerning intubation performance, Group LA had a significantly shorter intubation time compared to Group *T* and required significantly fewer rescue interventions (Table 3).

**TABLE 2:** Airway reflex incidence and intubation attempts in the studied groups.

**Parameter**		**Group LA (*n* = 40)**	**Group *T* (*n* = 40)**	**p** **value**	**Significance**

Cough/gag reflex incidence	No	32 (80.0%)	15 (37.5%)	*χ* ^2^:13.204, *p* < 0.001	HS
	Yes	8 (20.0%)	25 (62.5%)		

Intubation attempts	I	39 (97.5%)	29 (72.5%)	*χ* ^2^:7.941, *p* = 0.005	HS
	II	1 (2.5%)	11 (27.5%)		

*Note:* *χ* ^2^: chi‐square.

Abbreviation: HS, highly significant.

**TABLE 3:** Intubation performance in groups studied.

**Parameter**		**Group LA (*n* = 40)**	**Group *T* (*n* = 40)**	**p** **value**	**Significance**

Intubation time (s)	Mean ± SD	124.00 ± 23.68	201.35 ± 39.80	*t*:10.564, *p* < 0.001	HS

Rescue interventions	No	39 (97.5%)	22 (55.0%)	*χ* ^2^:17.670, *p* < 0.001	HS
	Yes	1 (2.5%)	18 (45.0%)		

*Note:* t: t‐test; *χ* ^2^: chi‐square.

Abbreviations: HS, highly significant; SD, standard deviation.

**FIGURE 5:**
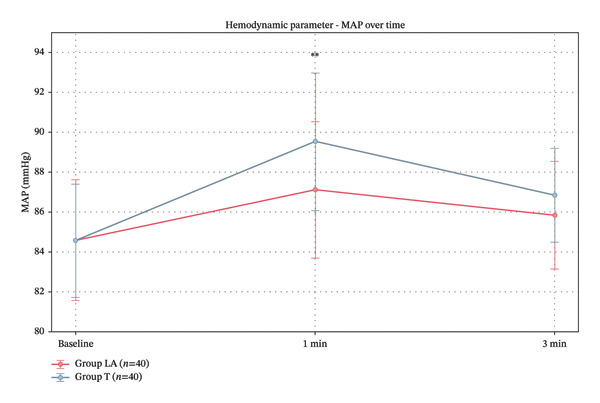
MAP in studied groups.

According to mean arterial pressure measurements, both groups had similar baseline MAP values. At 1 min post‐intubation, Group LA showed significantly lower MAP compared to Group *T*, while the difference at 3 min was not significant (Table 4) (Figure 5).

**TABLE 4:** Measurements of mean arterial blood pressure in groups studied.

**Parameter**		**Group LA (*n* = 40)**	**Group *T* (*n* = 40)**	**p** **value**	**Significance**

MAP baseline (mmHg)	Mean ± SD	84.60 ± 3.02	84.58 ± 2.85	*t*:0.038, *p* = 0.970	NS
MAP 1 min (mmHg)	Mean ± SD	87.12 ± 3.43	89.53 ± 3.45	*t*:3.121, *p* = 0.003	HS
MAP 3 min (mmHg)	Mean ± SD	85.85 ± 2.70	86.85 ± 2.35	*t*:1.766, *p* = 0.081	NS

*Note: t*: *t*‐test.

Abbreviations: HS, highly significant; MAP, mean arterial pressure; NS, nonsignificant; SD, standard deviation.

**FIGURE 6:**
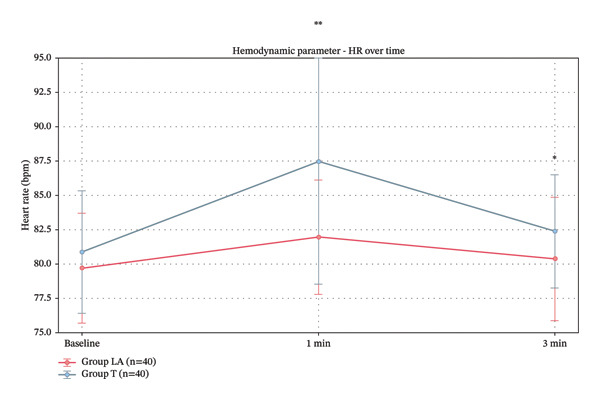
HR baseline in studied groups.

Regarding HR measurements, baseline values were comparable between Group LA and Group *T*. At 1 min post‐intubation, Group LA had a significantly lower HR than Group *T*, and this difference remained statistically significant at 3 min (Table 5) (Figure 6).

**TABLE 5:** HR in the studied groups.

**Parameter**		**Group LA (*n* = 40)**	**Group *T* (*n* = 40)**	**p** **value**	**Significance**

HR baseline (bpm)	Mean ± SD	79.72 ± 4.00	80.88 ± 4.47	*t*:1.213, *p* = 0.229	NS
HR 1 min (bpm)	Mean ± SD	81.97 ± 4.18	87.45 ± 8.89	*t*:3.525, *p* = 0.001	HS
HR 3 min (bpm)	Mean ± SD	80.38 ± 4.50	82.38 ± 4.13	*t*:2.071, *p* = 0.042	S

*Note: t*: *t*‐test.

Abbreviations: HR, heart rate; HS, highly significant; NS, nonsignificant S, significant; SD, standard deviation.

**FIGURE 7:**
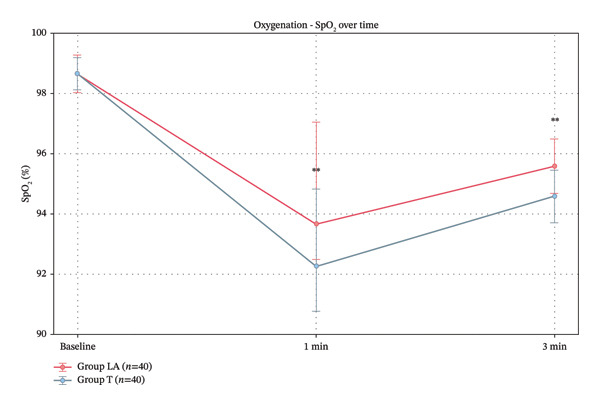
SpO_2_ baseline in studied groups.

In terms of oxygenation, baseline SpO_2_ levels were identical between Group LA and Group T. At 1 min post‐intubation, Group LA had significantly higher SpO_2_ than Group *T*, and this difference remained significant at 3 min (Table 6) (Figure 7).

**TABLE 6:** Oxygenation—SpO_2_ in the studied groups.

**Parameter**		**Group LA (*n* = 40)**	**Group *T* (*n* = 40)**	**p** **value**	**Significance**

SpO_2_ baseline (%)	Mean ± SD	98.65 ± 0.62	98.65 ± 0.53	*t*:0.000, *p* = 1.000	NS
SpO_2_ 1 min (%)	Mean ± SD	93.65 ± 1.17	92.25 ± 1.48	*t*:4.697, *p* < 0.001	HS
SpO_2_ 3 min (%)	Mean ± SD	95.58 ± 0.90	94.58 ± 0.87	*t*:5.034, *p* < 0.001	HS

*Note:* SpO_2_: peripheral oxygen saturation; t: *t*‐test.

Abbreviations: HS, highly significant; NS, nonsignificant; SD, standard deviation.

In terms of complications, Group *T* showed increased incidence of laryngospasm, sore throat, voice change, and oral injuries compared to Group LA. No difference in LA toxicity was observed, with no cases reported in either group. Nevertheless, these changes were statistically nonsignificant (Table 7).

**TABLE 7:** Complications in the studied groups.

**Parameter**		**Group LA (*n* = 40)**	**Group *T* (*n* = 40)**	**p** **value**	**Significance**

Laryngospasm	*n* (%)	1 (2.5%)	6 (15.0%)	*χ* ^2^:2.505, *p* = 0.113	NS
Sore throat	*n* (%)	6 (15.0%)	13 (32.5%)	*χ* ^2^:2.485, *p* = 0.115	NS
LA toxicity	*n* (%)	0 (0%)	0 (0%)	*χ* ^2^:0.000, *p* = 1.000	NS
Dysphagia	*n* (%)	38 (95.0%)	35 (87.5%)	*χ* ^2^:0.626, *p* = 0.429	NS
Voice change	*n* (%)	3 (7.5%)	7 (17.5%)	*χ* ^2^:1.029, *p* = 0.310	NS
Oral injuries	*n* (%)	1 (2.5%)	5 (12.5%)	*χ* ^2^:1.622, *p* = 0.203	NS

*Note:* *χ* ^2^: chi‐square.

Abbreviations: LA, local anesthetic; NS, nonsignificant.

Using VAS and patient recall, patient comfort was higher in Group *T*, with a statistically significant difference between groups (Table 8).

**TABLE 8:** Patient comfort and experience in the studied groups.

**Parameter**		**Group LA (*n* = 40)**	**Group *T* (*n* = 40)**	**p** **value**	**Significance**

VAS	Mean ± SD	5.55 ± 0.71	7.67 ± 0.57	*t*:14.683, *p* < 0.001	HS
Recall	*n* (%)	0 (0.0%)	9 (22.5%)	*χ* ^2^:8.013, *p* = 0.005	HS

*Note:* *χ* ^2^: chi‐square; t: *t*‐test.

Abbreviations: HS, highly significant; SD, standard deviation; VAS, Visual Analog Scale.

## 3. Discussion

Obesity significantly increases the risk of difficult airway management due to anatomical and physiological changes, causing higher complication rates during anesthesia, including difficult intubation and rapid desaturation [12].

The current study reveals that no significant differences were noted between Group *T* and Group LA regarding demographic data or airway assessment parameters, such as age, sex, BMI, Mallampati score, or TMD measurements. This consistency in baseline characteristics ensured attributing the noted outcome differences to intervention rather than confounding factors, thereby reinforcing the internal validity of the study. The alignment in demographic data is crucial in airway management studies, as factors like BMI and airway classification have been widely recognized as influential predictors of difficult intubation outcomes.

This observation aligns closely with the findings of Ramkumar et al. [13], who also emphasized the importance of maintaining balanced baseline characteristics when comparing airway block techniques. Similarly, El Deek et al. [7] and Magadum et al. [12] reiterated the critical need for ensuring comparability of study groups to avoid skewed results, particularly in studies evaluating nerve block efficacy in challenging airway scenarios.

In terms of airway reflex suppression, Group LA, which received ultrasound‐guided airway nerve blocks, exhibited a higher first‐attempt intubation success rate and a significantly lower incidence of cough and gag reflex compared to Group *T*. These results align with Mohanta et al.’s [11] work, demonstrating that ultrasound‐guided airway blocks led to superior suppression of airway reflexes and higher patient comfort compared to nebulized lignocaine.

Additionally, Zheng et al. [14] confirmed in their meta‐analysis that the use of airway nerve blocks, particularly the SLN block, provides more stable conditions for awake intubation and enhances the likelihood of first‐pass success. Nevertheless, Gostelow and Yeow [4] offered a critical perspective, noting that while ultrasound‐guided nerve blocks are effective, they require a high level of operator skill, and block failure remains possible in inexperienced hands. This underscores the need for comprehensive training and ongoing competency assessment in ultrasound‐guided airway management to ensure patient safety and procedural success.

This study revealed that Group LA had significantly shorter intubation times and required fewer rescue interventions compared to Group T. This observation conforms with Sun et al.’s [15] findings, reporting enhanced intubation conditions and reduced intubation time when an ultrasound‐guided SLN block is used for obese patients under bariatric surgery. This reduced need for rescue interventions also underscores nerve blocks’ superiority in maintaining airway patency and facilitating smoother intubation pathways.

Conversely, Krause et al. [16] highlighted that the successful execution of these blocks is highly dependent on the operator’s proficiency and that the technique, while effective, does have a learning curve. Supporting our findings, Sun et al. [15] and Mohanta et al. [11] also demonstrated improved intubation efficiency and reduced procedure times with ultrasound‐guided airway blocks, particularly in obese patients, where airway management is inherently more challenging. Similarly, Waheed et al. [17] emphasized the value of these techniques in optimizing intubation conditions and reducing airway trauma in such high‐risk populations. However, Iida et al. [18] and Malta et al. [19] advised caution, noting that while airway nerve blocks improve intubation conditions, their efficacy is maximized when used in conjunction with strong airway management skills and situational awareness.

Group LA demonstrated better hemodynamic stability with lower MAP and HR at 1 min post‐intubation, though these differences became nonsignificant at 3 min. Our findings are consistent with Wang et al. [20], demonstrating that SLN block effectively mitigates pressor responses during intubation more efficiently than topical anesthesia.

Furthermore, our findings corroborate the work of Terkawi et al. [21], who stressed that ultrasound‐guided airway nerve blocks offer superior control over hemodynamic parameters during airway manipulation, particularly in patients with anticipated difficult airways. However, Chaudhary [22] highlighted the need for caution when these techniques are applied to high‐risk populations, including those having cardiovascular compromise, due to the potential for exaggerated parasympathetic responses and sympathetic suppression, which could precipitate bradycardia or hypotension if not carefully monitored.

Patients in Group LA maintained significantly higher oxygen saturation levels post‐intubation and experienced fewer desaturation episodes and airway‐related complications such as laryngospasm and sore throat. These findings reflect the ability of ultrasound‐guided airway nerve blocks to minimize airway stimulation, maintain oxygenation, and reduce procedural‐related trauma, which is critical during awake or difficult intubation scenarios. These results are supported by Ramkumar et al. [13] and Okada et al. [23], who also demonstrated that ultrasound‐guided airway nerve blocks reduce airway stimulation and associated complications, leading to improved patient safety and procedural comfort.

Moreover, Zhipeng et al. [24] reported that desaturation episodes and sore throat postoperatively were significantly reduced by blocking the internal branch of the SLN, corroborating our observations.

Zhang et al. [25] found that the use of topical anesthesia, particularly for obese patients, was combined with heightened desaturation episodes, highlighting those limitations of topical approaches in challenging airway management scenarios, which was consistent with this study. These findings emphasize the superiority of nerve blocks over traditional topical techniques, especially in patients at high risk for desaturation.

Conversely, Mohanta et al. [11] proposed that nebulized lignocaine may still have a role in selected patients, particularly those who are highly cooperative and can tolerate awake procedures without significant airway hyperreactivity. They emphasized that in cases where nerve blocks are contraindicated or not feasible, nebulization remains a viable, albeit less effective, alternative.

In this study, patient‐reported comfort, as assessed by VAS scores, and the absence of procedural recall were significantly better in Group LA compared to Group *T*, reflecting higher patient satisfaction and lower procedural stress levels. Those results align with Huang et al. [26], demonstrating that patient comfort and cooperation while awake were significantly enhanced using ultrasound‐guided SLN block for endotracheal intubation, leading to smoother procedures and greater overall satisfaction.

Similarly, Liao et al. [27] confirmed the benefits of airway nerve blocks in improving patient tolerance during rigid bronchoscopy, highlighting their utility in procedures that require patients to maintain spontaneous breathing while ensuring comfort. However, despite these advantages, Gostelow and Yeow [4] cautioned that patient education, reassurance, and thorough psychological preparation remain essential, even when airway blocks are applied.

Additionally, Tasdemir et al. [3] and Zhang et al. [28] reiterated the critical role of technique and airway management approach in mitigating anatomical difficulties, reinforcing that a key component in difficult airway algorithms is skillfully using ultrasound‐guided nerve blocks. However, Krause et al. [16] emphasized that despite the clear benefits of ultrasound‐guided airway nerve blocks, they should not be relied upon as standalone interventions. They stressed that these blocks must be integrated into a comprehensive airway management strategy that includes contingency plans, adjunct techniques, and readiness for advanced airway interventions if required. This holistic approach ensures that the limitations of nerve blocks are recognized, and patient safety is preserved throughout the procedure.

While the present study demonstrated the superiority of ultrasound‐guided airway nerve blocks over topical lidocaine spray across all measured outcomes, topical anesthesia remains a clinically relevant alternative in settings where ultrasound guidance or the requisite operator expertise is unavailable. Prior literature acknowledges that topical lidocaine spray offers a rapid onset, ease of application, and acceptable tolerability and that it can provide safe plasma concentrations with limited adverse effects [29]. However, these attributes must be interpreted within the context of our findings: In obese patients with anticipated difficult intubation, topical spray was associated with significantly higher rates of cough/gag reflex, longer intubation times, greater hemodynamic perturbation, and inferior oxygenation profiles. Therefore, when feasible, ultrasound‐guided airway nerve blocks should be the preferred technique in this high‐risk population, while topical anesthesia may be reserved as a contingency in resource‐limited or time‐critical scenarios.

## 4. Conclusion

This study demonstrated that ultrasound‐guided airway nerve blocks provided shorter intubation time, significantly superior airway conditions, and more stable hemodynamic and oxygenation profiles compared to topical lidocaine spray for obese patients anticipated to face difficult intubation undergoing bariatric surgery. Patients in the nerve block group experienced fewer airway‐related complications, reduced need for rescue interventions, and higher overall comfort with no recall of the procedure.

## 5. Limitations and Recommendations

An anesthesiologist’s experience was mandatory for the effectiveness of the block, so training programs to enhance skills and patient safety are recommended. A single center with a limited number was the place for conducting this study; hence, it is recommended to carry out additional large‐scale, multicenter studies for validating these findings and exploring the wider airway nerve blocks’ applicability to other high‐risk surgical populations.

A key limitation is the use of a standardized conscious sedation protocol (dexmedetomidine 0.7 mcg/kg/hr infusion plus ketamine 0.5 mg/kg IV) in both groups. While uniformly administered to ensure patient cooperation and the ethical conduct of the awake procedure, both agents possess independent pharmacological properties relevant to the primary outcomes. Dexmedetomidine attenuates the hemodynamic response to airway manipulation and may mildly suppress airway reactivity via central alpha‐2 agonism, while sub‐anesthetic ketamine may reduce cough and gag sensitivity through NMDA receptor modulation. Although the identical protocol preserves internal comparability, it prevents exclusive attribution of observed benefits to the airway anesthesia technique alone and limits generalizability to settings employing different or no sedation. Future studies with varying or no sedation backgrounds would help delineate the independent contributions of airway nerve blocks versus topical anesthesia.

The single‐blinded design, while pragmatically necessary given the visible procedural differences between the two techniques, introduces a risk of performance and detection bias for subjective outcomes, including cough/gag reflex incidence and patient comfort scores. Although a dedicated independent observer performed outcome assessments using binary criteria, formal blinding verification was not conducted, and the possibility that operator awareness influenced procedural behavior or assessment cannot be entirely excluded.

## Author Contributions

Dr. Reham M. Hashim: design of the work, provision of materials, patients and resources, statistical expertise, critical revision of the article, and final approval of the article. Dr. Mohamed S. M. Zaki: analyzing and drafting the data, writing the article, provision of materials, patients, and resources, and final approval of the article, administrative, technical, or logistic support. Dr. David B. N. Elagiby: provision of materials, patients and resources, analysis and interpretation, critical revision of the article, and final approval of the article. Dr. Islam A. A. Taher: literature search, critical revision of the article, data collection, and writing the article.

## Funding

This study was self‐funded.

## Disclosure

All authors have participated, read, and approved the manuscript.

## Ethics Statement

It was taken from the ethical committee of the Faculty of Medicine, Ain Shams University (FMASU MS 120/2025). All patients signed written informed consent before inclusion and were informed about the details of the procedure.

## Consent

Please see the ethics statement.

## Conflicts of Interest

The authors declare no conflicts of interest.

## Data Availability

The datasets used and/or analyzed during the current study are available from the corresponding author on reasonable request.
